# Predictive value of the monocyte-to-high-density lipoprotein cholesterol ratio in atrial fibrillation: a meta-analysis

**DOI:** 10.3389/fcvm.2026.1620841

**Published:** 2026-02-02

**Authors:** Xiangzhu Meng, Yuhang Wen, Xiangying Wang, Xiaolei Yang, Lianjun Gao

**Affiliations:** 1 Department of Cardiology, Institute of Cardiovascular Diseases, The First Affiliated Hospital of Dalian Medical University, Dalian, China; 2 Department of Rehabilitation, Jiangxi Province Hospital of Integrated Chinese and Western Medicine, Nanchang, China

**Keywords:** atrial fibrillation, biomarker, inflammation, meta-analysis, monocyte-to-high-density lipoprotein cholesterol ratio

## Abstract

**Background:**

The monocyte-to-high-density lipoprotein cholesterol ratio (MHR) has emerged as a novel biomarker for cardiovascular outcomes. However, its role in atrial fibrillation (AF) remains unclear. This meta-analysis aimed to evaluate the diagnostic efficacy of MHR in predicting AF risk.

**Methods:**

We systematically searched PubMed, Embase, and Web of Science up to March 20, 2025. The primary outcome was to assess the diagnostic accuracy of MHR for predicting AF using summary receiver operating characteristic (SROC) curve analysis. The secondary outcome was to explore the relationship between MHR and AF risk. Pooled odds ratio (OR), sensitivity, specificity, and area under the curve (AUC) were calculated.

**Results:**

A total of 13 studies comprising 5,499 participants were included. Elevated MHR was independently associated with an increased AF risk (OR = 1.21; 95% CI, 1.11–1.31; *P* < 0.001). The pooled sensitivity and specificity were 0.85 (95% CI, 0.71–0.93) and 0.68 (95% CI, 0.60–0.75), yielding an area under the SROC curve of 0.80 (95% CI, 0.76–0.83). Subgroup analyses revealed significant diagnostic performance variations by AF phenotype: MHR had the highest sensitivity (0.91; 95% CI 0.74–1.00) and AUC (0.94; 95% CI 0.91–0.96) in non-procedural AF, followed by post-ablation recurrence (sensitivity = 0.86, AUC = 0.83) and new-onset AF (sensitivity = 0.80, AUC = 0.83). Large-sample studies (>600) showed lower sensitivity (0.71 vs. 0.90) but higher specificity (0.78 *vs.* 0.60) than small-sample studies (≤600). No significant publication bias was detected (*p* = 0.45).

**Conclusions:**

MHR demonstrates moderate diagnostic accuracy for AF risk prediction and is better suited as a screening or complementary biomarker than a standalone diagnostic tool. Its diagnostic performance varies significantly by AF phenotype and clinical context. Given the limited number of studies, significant heterogeneity, and unstandardized MHR cut-offs, large-scale prospective studies with standardized protocols are warranted to validate these findings and facilitate targeted clinical application.

**Systematic Review Registration:**

https://www.crd.york.ac.uk/prospero/, identifier CRD420251030225.

## 1 Introduction

Atrial fibrillation (AF), characterized by rapid and irregular activation of the atrium, is one of the most common clinical arrhythmias, and its incidence is increasing, partly due to the global ageing population (1). The diagnosis of AF primarily relies on electrocardiograms (ECGs). The foundation of AF management includes heart rate control, anticoagulation, and rhythm control for patients whose symptoms are significantly affected by the condition. Despite advances in diagnosis and treatment, the incidence of AF remains high. By 2050, the number of patients affected by AF is projected to increase 2.5-fold, with more than half aged 80 or older (2). In the US, 6–12 million people will suffer from AF by 2050, while in Europe, 17.9 million will be affected by 2060 (3). AF not only leads to impaired cardiac function and increases the risk of heart failure, stroke, and mortality, but it also significantly affects patients' quality of life, thereby placing a substantial burden on healthcare systems and the economy (4). Early identification of high-risk populations for AF and timely implementation of interventions are essential to reduce its incidence and associated complications and to uncover potential therapeutic targets. Therefore, the discovery of effective biomarkers that can predict AF is of great importance.

The pathophysiological mechanisms underlying AF are intricate and multifactorial, involving inflammation, oxidative stress, apoptosis, fibrosis, and genetic factors. These processes contribute to structural and electrophysiological alterations during atrial remodeling (5, 6). Inflammation plays a crucial role in forming the atrial substrate, facilitating both structural and electrical remodeling by releasing pro-inflammatory cytokines and other inflammatory molecules, thereby heightening susceptibility to AF (7). Furthermore, inflammation disrupts calcium homeostasis and impairs connexin function, which are linked to AF triggers and heterogeneous atrial conduction (8). Oxidative stress results from an imbalance between reactive oxygen species (ROS) production and the body's antioxidant defenses. This condition fosters a pro-inflammatory state, leading to tissue damage, cardiac remodeling, and AF progression (9). Consequently, numerous studies have explored the relationship between inflammatory biomarkers and AF. A variety of inflammatory biomarkers, such as high-sensitivity C-reactive protein (hs-CRP), interleukin-2 (IL-2), interleukin-6 (IL-6), and tumor necrosis factor-alpha (TNF-α), have been associated with both the risk and progression of AF (10, 11).

The monocyte-to-high-density lipoprotein cholesterol ratio (MHR) is a novel biomarker reflecting inflammation, oxidative stress, and metabolic syndrome. First proposed by Kanbay et al. in 2014, MHR has been found to be associated with adverse cardiovascular events in patients with chronic kidney disease (12). Since then, MHR has been extensively studied, particularly in the context of risk stratification and prognosis for cardiovascular diseases (13). Monocytes originate from hematopoietic stem cells in the bone marrow and are a major component of the human immune system. Activated monocytes can stimulate the production of inflammatory cytokines and pro-oxidants, thereby contributing to endothelial injury and atherosclerosis formation (14, 15). High-density lipoprotein cholesterol (HDL-C) promotes reverse cholesterol transport, enabling the removal of excess cholesterol from peripheral tissues to the liver. HDL-C exhibits anti-inflammatory, antioxidant, antithrombotic, and anti-atherosclerotic properties, all of which positively influence cardiovascular outcomes (16). Elevated MHR levels indicate greater systemic inflammation and immune activation. Combining monocyte and HDL-C parameters may provide more valuable information about inflammation and oxidative stress status than either parameter alone. Moreover, MHR stands out as a highly accessible biomarker in clinical settings due to its affordability and widespread availability. Interestingly, recent studies have highlighted the predictive role of MHR in AF development. However, there is no consensus on the capacity of MHR for AF prediction. We therefore conducted this systematic review and meta-analysis to evaluate whether MHR could serve as a predictive biomarker for AF and to inform clinical management.

## 2 Materials and methods

### 2.1 Data source and protocol registration

The present systematic review and meta-analysis were conducted in accordance with the Preferred Reporting Items for Systematic Reviews and Meta-Analyses (PRISMA) 2020 recommendations (17) and registered in PROSPERO (CRD420251030225). A literature search was performed across three databases (PubMed, Embase, and Web of Science) to identify relevant studies up to March 20, 2025. The search strategy was as follows: (“monocyte to HDL-cholesterol ratio” OR “monocyte/HDL-cholesterol ratio” OR “monocyte to high-density lipoprotein cholesterol ratio” OR “monocyte/high-density lipoprotein ratio” OR “monocyte/HDL ratio” OR “monocyte to HDL ratio” OR “MHR”) AND (“atrial fibrillation” OR “AF”). The included studies had no language or geographic restrictions. Additionally, we reviewed the reference lists of relevant original studies and major review articles to identify further relevant studies.

### 2.2 Study inclusion and exclusion criteria

Articles were included in this meta-analysis if they met the following criteria: (1) study type including randomized controlled trials, prospective or retrospective cohort studies, case-control studies, or cross-sectional studies; (2) reported the hazard ratio (HR) or odds ratio (OR) with their corresponding 95% confidence intervals (CIs) for the association between MHR and the risk of AF; (3) evaluated the diagnostic performance of MHR in AF; (4) provided available data to directly or indirectly calculate true positive (TP), false positive (FP), false negative (FN), and true negative (TN) values to construct the 2 × 2 table; (5) had a clearly established diagnosis of AF in accordance with current guideline definitions. Exclusion criteria were established as follows: (1) letters, editorials, conference presentations, studies without a control group, case reports, and non-human studies; (2) studies with inaccessible full text or missing data; (3) duplicate or overlapping publication; (4) studies with a high risk of bias or controversial design.

### 2.3 Data extraction and quality assessment

Two authors independently extracted the following data: name of the first author, year of publication, country, study design, sample sizes, age, gender, study periods year, MHR cut-off values, sensitivity, specificity, rates of postoperative AF, and follow-up period. Monocyte counts were standardized to cells/µL. For studies reporting HDL-C in mmol/L, we converted it to mg/dL using the conversion factor: 1 mmol/L = 38.67 mg/dL. The methodological quality of each included study was independently evaluated by two authors using the Quality Assessment of Diagnostic Accuracy Studies-2 (QUADAS-2) tool (18). The QUADAS-2 tool assesses four main domains: patient selection, index test, reference standard, and flow and timing. The risk of bias in each domain was assessed and categorized as high, low, or unclear. Any disagreements were resolved through discussion or consultation with a third reviewer. The quality assessment was conducted using Review Manager (Version 5.4). The green portion indicates compliance with the standard requirements, the red portion signifies non-compliance, and the yellow portion represents uncertainty.

### 2.4 Primary and secondary outcome

The primary outcome was to evaluate the diagnostic accuracy of MHR for predicting AF using summary receiver operating characteristic (SROC) curve analysis. The secondary outcome was to assess the pooled odds ratio (OR) for the association between MHR and AF risk.

### 2.5 Statistical analysis

All statistical analyses were conducted using STATA, version 14.0 (Stata Corporation, College Station, TX) and R software (version 4.5.0). Both Cochrane's Q test and the I2 statistic were used to assess heterogeneity across studies. A *P*-value < 0.10 or I^2^ > 50% indicated statistically significant heterogeneity. We chose the random-effects model to synthesize results when heterogeneity was significant; otherwise, the fixed-effects model was applied. When heterogeneity was present, meta-regression and subgroup analysis were used to explore its sources. Meta-Disc 1.4 software was used to assess threshold effects using the Spearman correlation coefficient between the logarithm of sensitivity and 1-specificity. When the *P*-value was < 0.05, the threshold effect was considered significant. Overall diagnostic accuracy was evaluated by plotting the summary receiver operating characteristic (SROC) curve. The area under the curve (AUC) was calculated as a measure of diagnostic performance. An AUC > 0.7 was considered a significant predictor of risk (19). When there was no evidence of a threshold effect, the bivariate random-effects model was applied to estimate pooled sensitivity, specificity, positive likelihood ratio (+LR), negative likelihood ratio (−LR), diagnostic score, and diagnostic odds ratio (DOR). Publication bias was assessed using the Deeks' funnel plot asymmetry test. In all analyses, a *P* value of 0.05 was employed to assess statistical significance.

## 3 Results

### 3.1 Search results and studies characteristics

**Figure 1:**
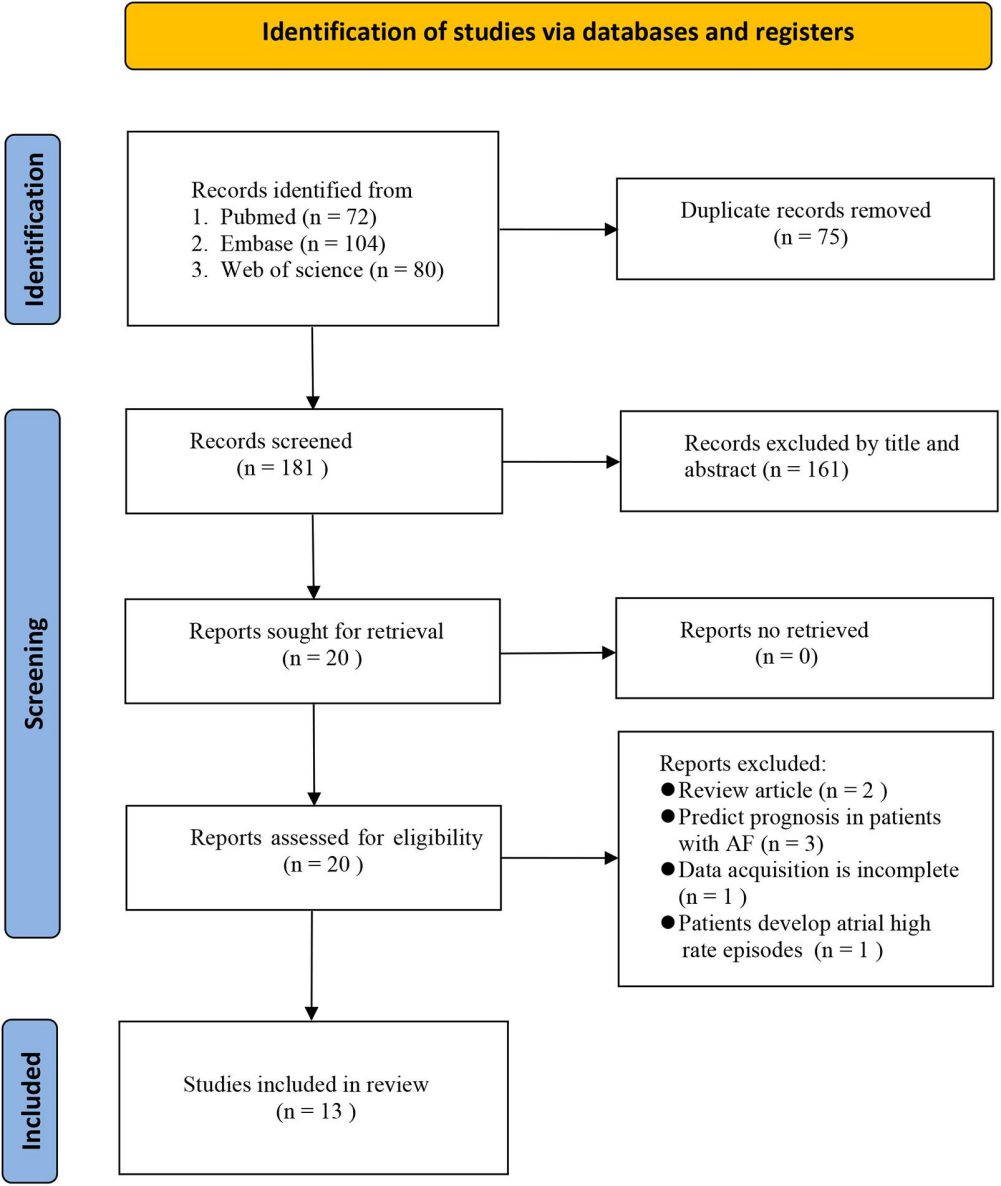
Flow diagram of study selection.

The literature screening flow diagram is shown in Figure 1. A total of 256 studies were retrieved from the three databases using the search strategy. We identified 75 duplicates and excluded them. The remaining 181 studies were screened by title and abstract to identify relevant studies. After excluding 161 studies that did not meet the inclusion criteria, 20 were eligible for comprehensive full-text review. Seven articles were excluded after full-text review for the following reasons: review articles (*n* = 2), prognostic prediction in patients with AF (*n* = 3), incomplete data acquisition (*n* = 1), and patients developing atrial high-rate episodes (*n* = 1). Finally, thirteen eligible studies involving 5,499 participants were included and analyzed (20–32).

**Figure 2:**
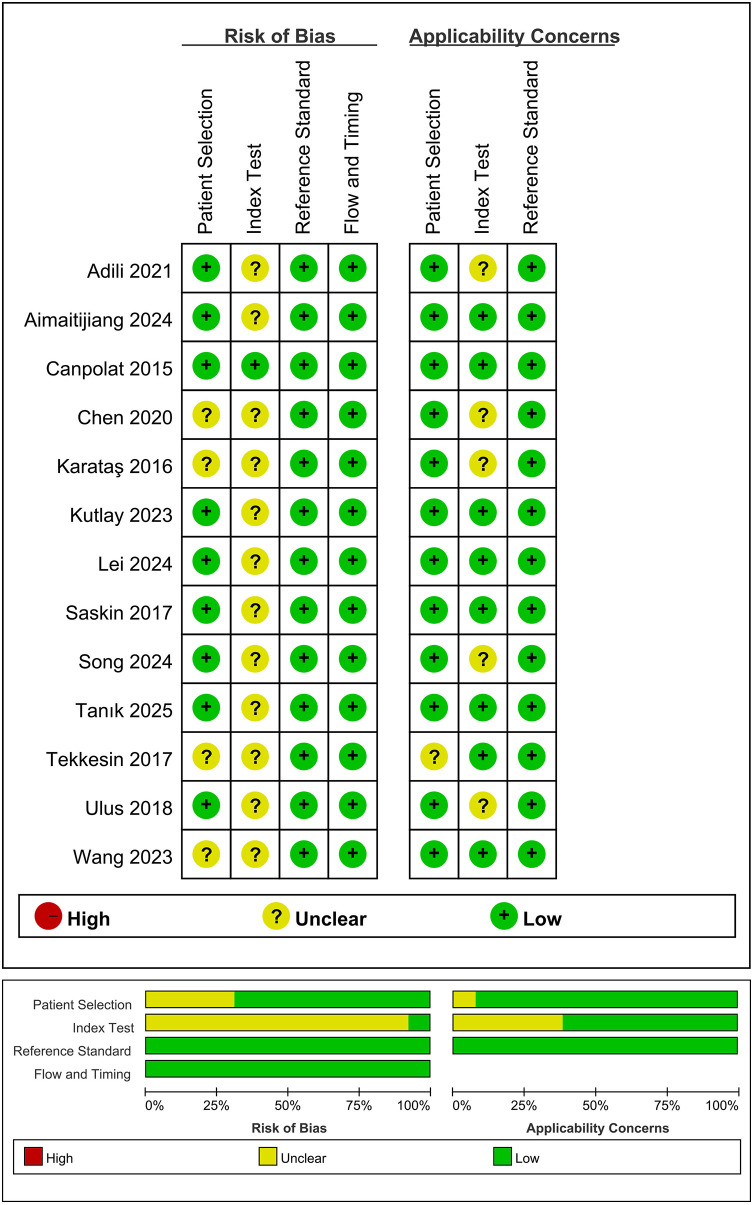
Quality assessment of included studies based on QUADAS-2 tool criteria.

The main characteristics and findings of the included studies are presented in Table 1. Sample sizes ranged from 131 to 817, and mean patient age ranged from 53.5 to 74.4 years; the overall proportion of males was high. Follow-up periods ranged from 3 to 25.1 months. Among the included studies, one study was prospective (20), and the remaining twelve were retrospective (21–32). Seven studies were conducted in Turkey (20–24, 28, 32), and six in China (25–27, 29–31). Five studies investigated the relationship between MHR and new-onset AF after coronary artery bypass grafting (CABG) and percutaneous coronary intervention (PCI) (21–24, 32). Six studies reported an association between MHR and AF recurrence after radiofrequency maze procedure and catheter ablation (20, 25, 26, 29–31). According to the QUADAS-2 results (Figure 2), all 13 included studies were of high quality. The index test was identified as the primary source of high risk, as the diagnostic cut-off values were based on predefined thresholds. In contrast, the other domains of the studies showed a lower risk of bias.

**Table 1:** Basic characteristics of studies included into the meta-analysis.

Author/year	Country	Study design	Study periods year	Study population	Patients (*n*)	Male (%)	Mean age years	Follow-up (month)	Rates of postoperative AF	Cut-off	Sensitivity	Specificity	AUC
Canpolat 2015	Turkey	Prospective	2010–2013	Patients with AF after cryoballoon ablation	402	227 (56.5%)	53.5 ± 10.9	20.6 ± 6	23.6%	11.48	85%	74%	0.85
Karataş 2016	Turkey	Retrospective	2009–2013	Patients with STEMI after PCI	621	464 (74.7%)	57 ± 11.9	22	64.4%	25.81	75%	80%	0.84
Saskin 2017	Turkey	Retrospective	2010–2014	After CABG	662	541 (81.7%)	60.9 ± 6.9	NA	23.1%	18.50	86.9%	72.5%	0.84
Tekkesin 2017	Turkey	Retrospective	2015	After CABG	311	205 (65.9%)	60.1 ± 8.7	NA	22.8%	8.55	NA	NA	0.84
Ulus 2018	Turkey	Retrospective	2016–2017	Patients with ACS after PCI	308	202 (65.6%)	74.4 ± 6.5	NA	17.5%	15.87	75.9%	65.0%	0.75
Chen 2020	China	Retrospective	2015–2018	Patients with AF after RFCA	125	69 (55.2%)	61.2 ± 9.3	25.1 ± 12.0	37.6%	NA	NA	NA	0.71
Adili 2021	China	Retrospective	2018–2019	Patients with AF after RF maze procedure	131	54 (41.2%)	60 (54–67)	3	53.4%	8.53	89%	54%	0.77
Wang 2023	China	Retrospective	2019–2022	AF patients and controls	817	504 (61.7%)	61.9 ± 9.8	NA	NA	16.94	42.7%	85.2%	0.60
Kutlay 2023	Turkey	Retrospective	2019–2021	AF patients and controls	241	115 (47.7%)	65.7 ± 9.9	NA	NA	15.00	100%	56%	0.80
Song 2024	China	Retrospective	2019–2021	Patients with AF after catheter ablation	438	268 (61.2%)	62 (54–66)	NA	26.3%	NA	NA	NA	0.64
Aimaitijiang 2024	China	Retrospective	2015–2018	Patients with AF after cryoablation	570	100 (17.5%)	66 ± 9.3	24	19.8%	7.50	76%	44%	0.60
Lei 2024	China	Retrospective	2020–2022	Patients with AF after RFCA	210	141 (67.1%)	54.4 ± 8.7	12	37.1%	8.68	93.59%	65.91%	0.83
Tanık 2025	Turkey	Retrospective	2017–2018	Patients with STEMI after PCI	663	572 (86.3%)	55.8 ± 12.9	NA	5.1%	26.54	70.59%	72.45%	0.77

AF, atrial fibrillation; STEMI, ST segment elevation myocardial infarction; ACS, acute coronary syndrome; PCI, percutaneous coronary intervention; CABG, coronary artery bypass grafting; RF, radiofrequency; RFCA, radiofrequency catheter ablation.

### 3.2 Meta-analysis

#### 3.2.1 The relationship between MHR and AF risk

**Figure 3:**
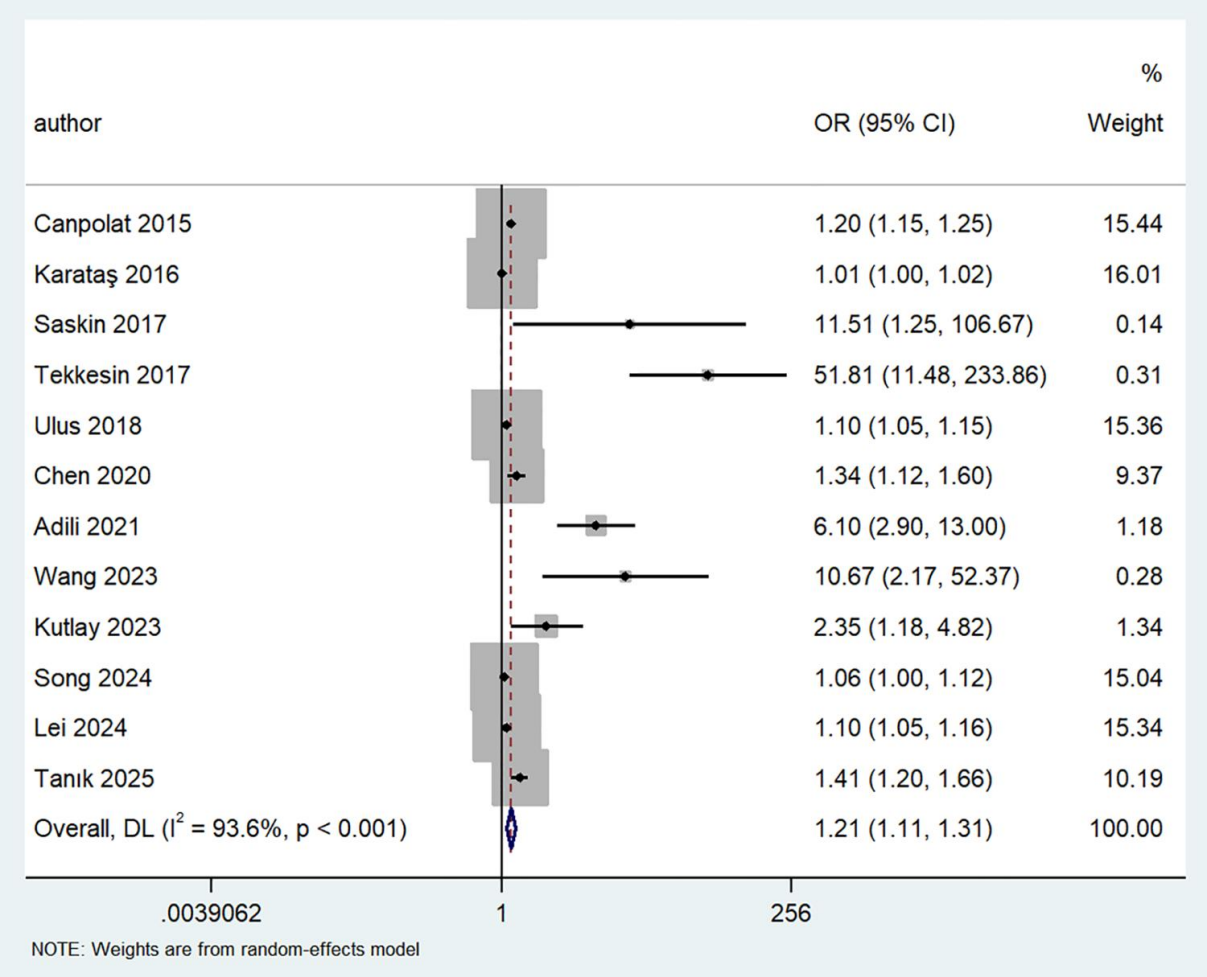
Forest plot showing the odds ratio for AF in patients with high vs. low MHR levels.

Twelve studies examined the association between MHR and AF risk, using a random-effects model (I^2^ = 93.6%, *P* < 0.001) to pool the effect size. The results showed a significant correlation between MHR and AF risk (OR = 1.21, 95% CI: 1.11–1.31, *P* < 0.001), indicating that MHR was an independent predictor of AF (Figure 3).

#### 3.2.2 Analysis of the threshold effect

In diagnostic meta-analyses, sources of heterogeneity include threshold and non-threshold effects. The SROC curve did not exhibit the typical “shoulder-arm” shape, and Spearman's correlation coefficient between sensitivity and log(1−specificity) was 0.6 (*P* = 0.067, *P* > 0.05), indicating no significant heterogeneity attributable to the threshold effect and supporting pooling of the data.

#### 3.2.3 Diagnostic efficacy of MHR in predicting AF

**Figure 4:**
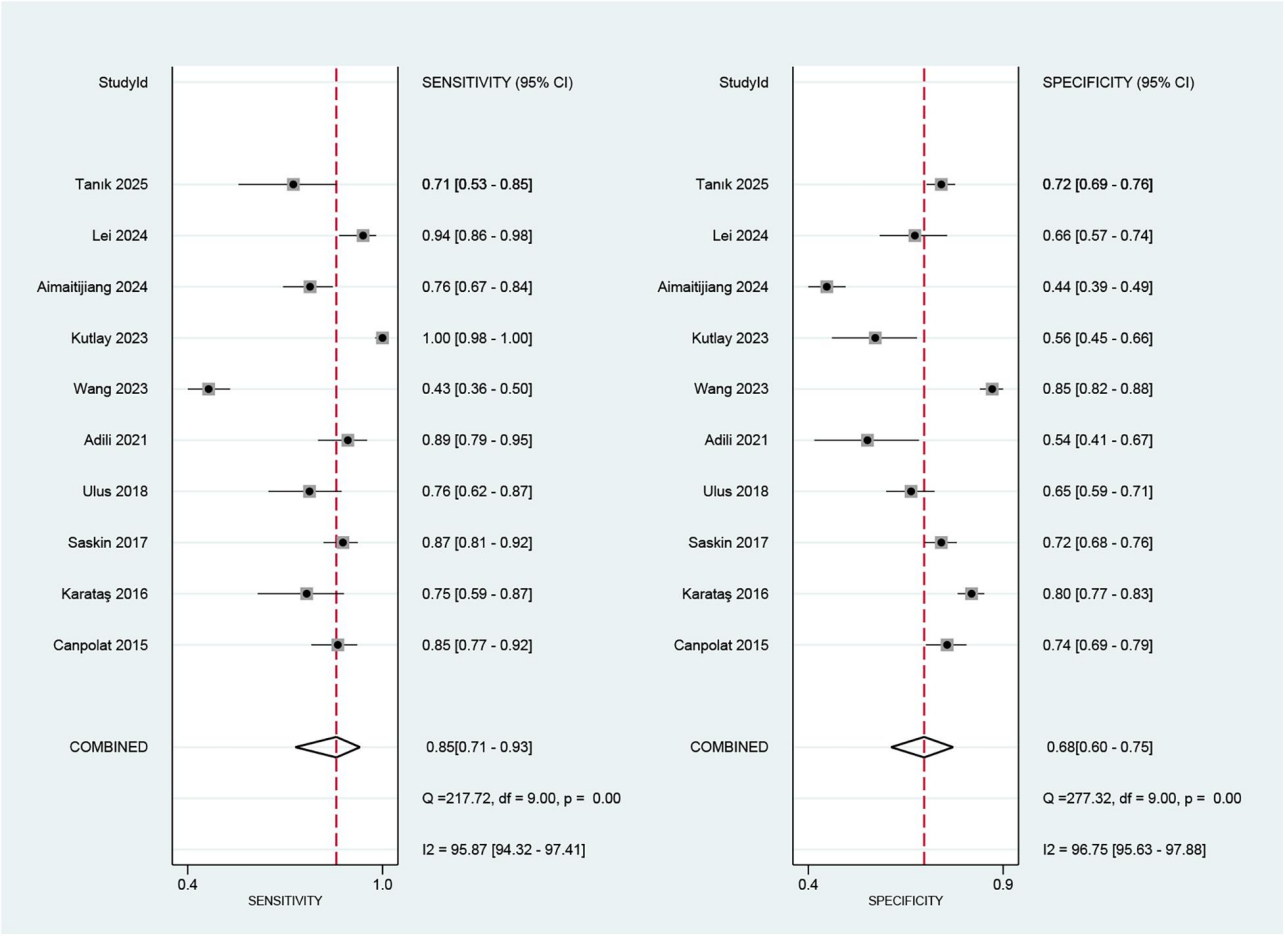
Forest plot depicting the combined sensitivity and specificity of the MHR in predicting AF.

**Figure 5:**
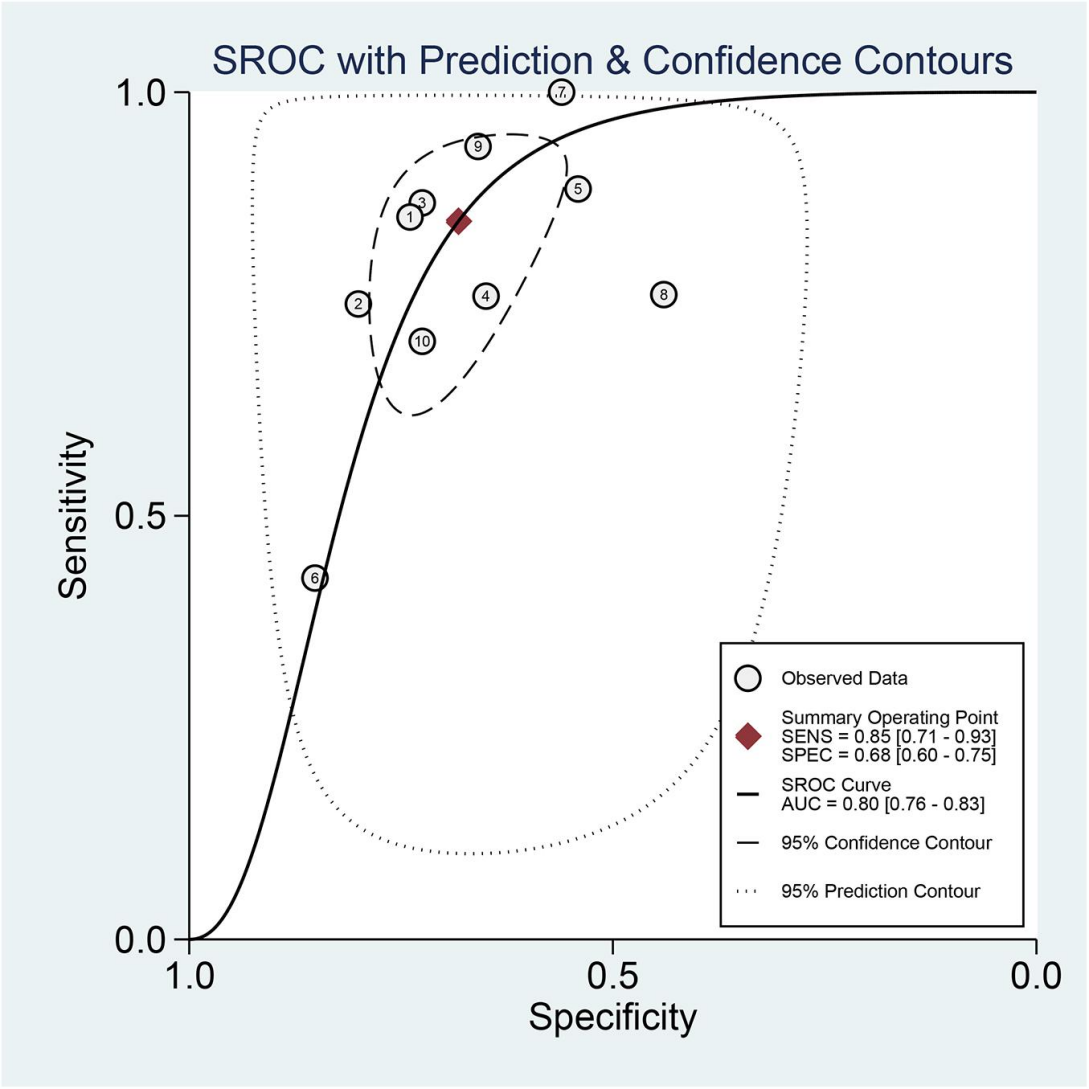
Analysis of SROC curve showing the predictive effectiveness of MHR concerning AF.

Regarding the diagnostic accuracy of MHR, the pooled sensitivity was 0.85 (95% CI: 0.71–0.93), and the specificity was 0.68 (95% CI: 0.60–0.75). Significant heterogeneity was observed across studies. The I^2^values were 95.87% (95% CI: 94.32–97.41) for sensitivity and 96.75% (95% CI: 95.63–97.88) for specificity (Figure 4). As shown in Supplementary Figure S1, the +LR and −LR were reported as 2.67 (95% CI: 2.16–3.30) and 0.22 (95% CI: 0.12–0.42), respectively. The diagnostic score and DOR presented in (Supplementary Figure S2) were 2.48 (95% CI: 1.78–3.18) and 11.94 (95% CI: 5.90–24.17), respectively. The SROC curve in Figure 5 showed an AUC of 0.80 (95% CI: 0.76–0.83), suggesting moderate diagnostic accuracy.

#### 3.2.4 Fagan nomogram for post-test probabilities

**Figure 6:**
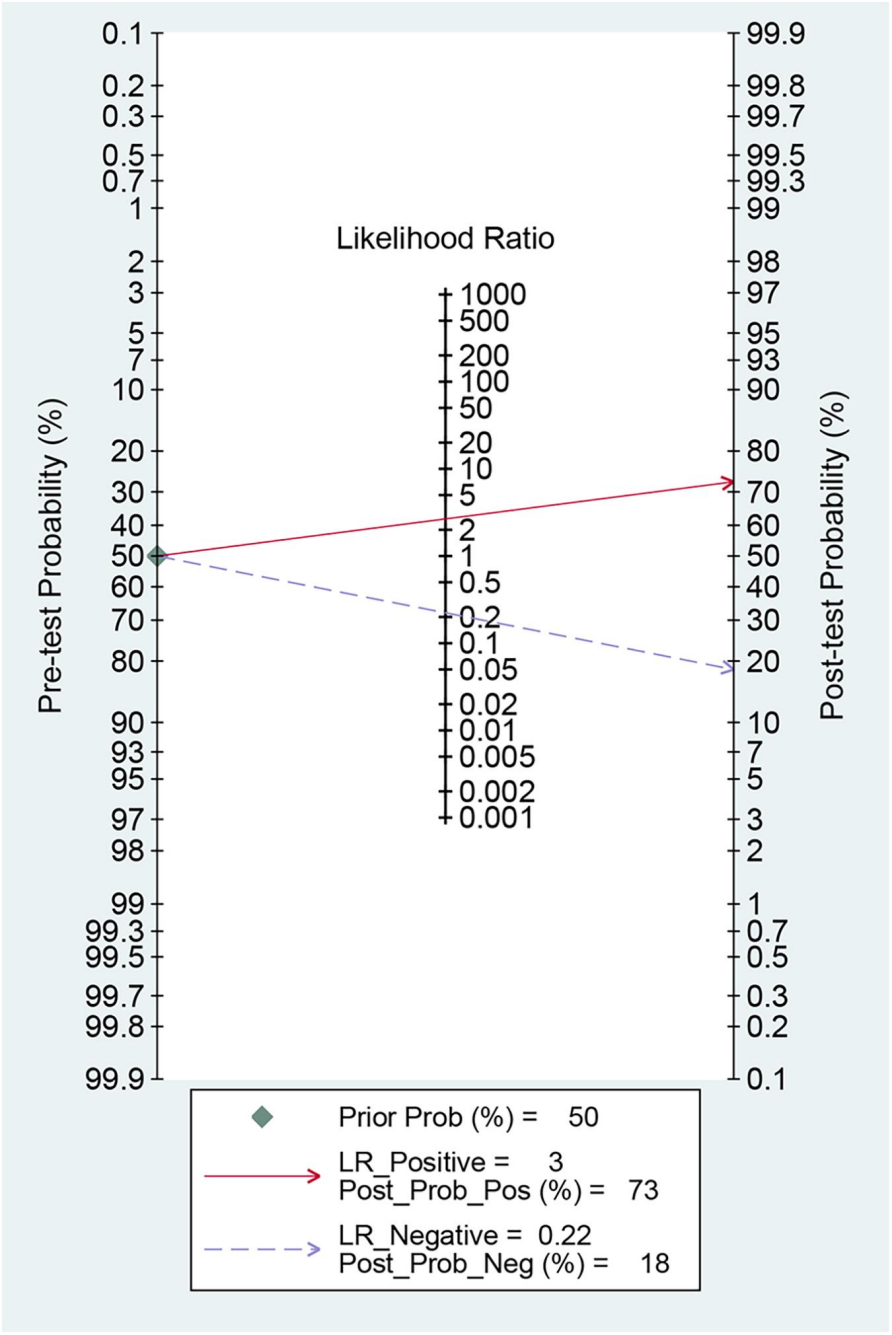
Fagan nomogram for assessing the clinical utility of MHR for predicting AF.

Fagan's nomogram is a graphical tool used to evaluate the clinical utility of diagnostic tests by quantifying post-test probabilities. The current analysis yielded a +LR of 3.00 and a −LR of 0.22. Consequently, with a pre-test probability set at 50%, the post-test probability of AF increased to 73% for a positive MHR test; conversely, it decreased to 18% for a negative test. These results indicate that a positive MHR test significantly elevates the likelihood of AF to 73%, while a negative test reduces this probability to 18% relative to the pre-test probability (Figure 6).

#### 3.2.5 Meta-regression and subgroup analysis

**Figure 7:**
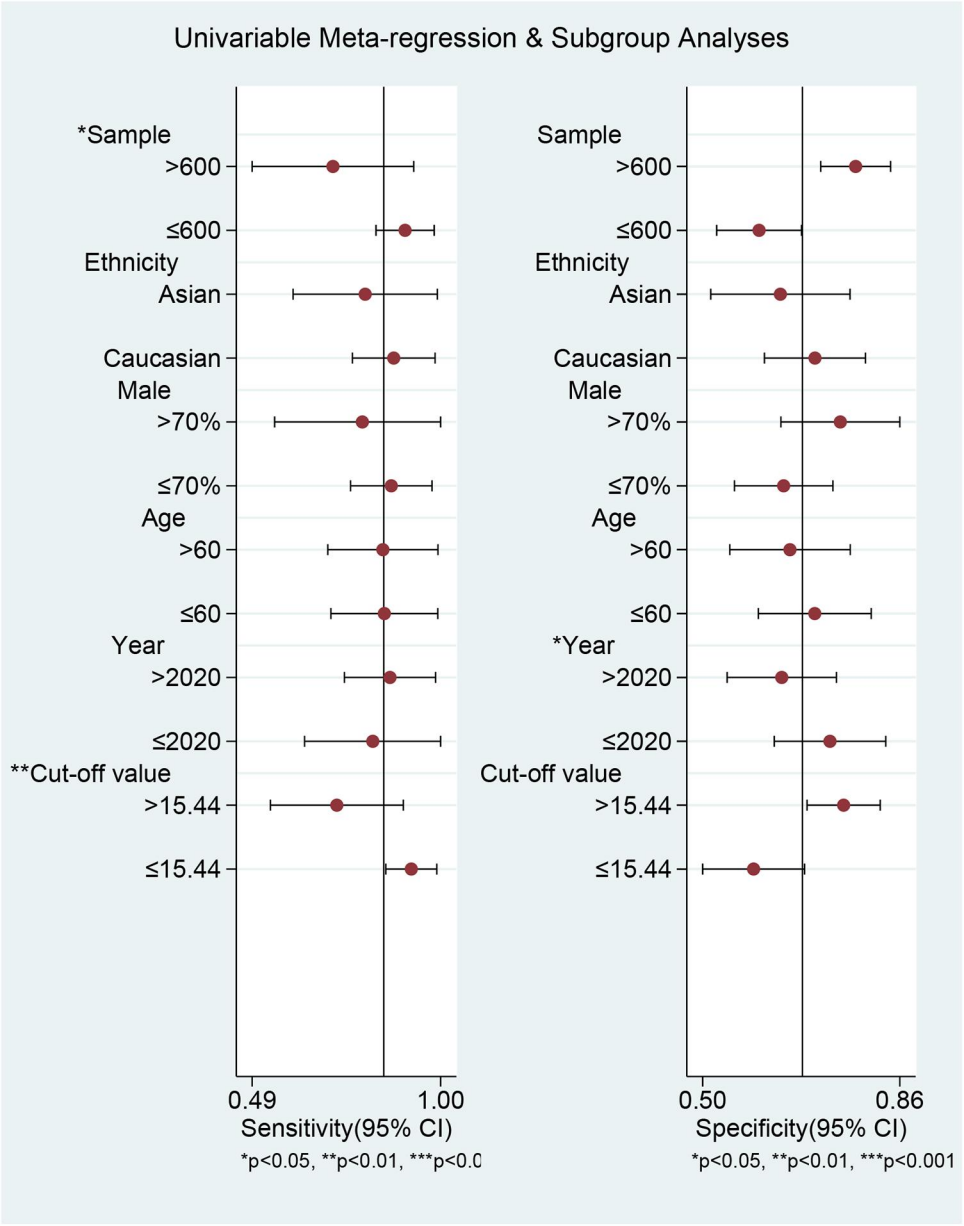
The results of meta-regression and subgroup analyses.

We conducted meta-regression and subgroup analyses to explore sources of heterogeneity in sensitivity and specificity across the included studies. The variables included were sample size, ethnicity, mean age, proportion of males, article publication year, cut-off value, and study population (Figure 7; Table 2). We found that sample size and cut-off value significantly contributed to heterogeneity in sensitivity but not in specificity. In contrast, publication year significantly affected heterogeneity in specificity but not in sensitivity. The joint model results indicated that sample size and cut-off value subgroups significantly affected heterogeneity in both sensitivity and specificity. When stratified by sample size, pooled sensitivity was higher in small-sample studies (≤600) at 0.90 (95% CI: 0.83–0.98) than in large-sample studies (>600) at 0.71 (95% CI: 0.49–0.93), whereas specificity (0.60 vs. 0.78) and AUC (0.74 *vs.* 0.82) showed the opposite trend. Ethnicity stratification revealed that studies involving Caucasian populations had higher sensitivity [0.87 (95% CI: 0.76–0.99)], specificity [0.71 (95% CI: 0.61–0.80)], and AUC [0.79 (95% CI: 0.75–0.82)] than those involving Asian populations [sensitivity: 0.80 (95% CI: 0.60–0.99), specificity: 0.64 (95% CI: 0.51–0.77), AUC: 0.77 (95% CI: 0.73–0.80)]. Stratification by male proportion showed higher sensitivity in the lower proportion group (≤70%: 0.87 vs. >70%: 0.79), albeit with lower specificity (0.65 vs. 0.75) and AUC (0.78 vs. 0.83). Stratification by age showed identical sensitivity [0.85 (95% CI: 0.70–0.99)] in older and younger patients, but superior specificity (>60 years: 0.71 vs. ≤60 years: 0.66) and AUC (>60 years: 0.84 vs. ≤60 years: 0.77) in older patients. Studies published after 2020 had slightly higher sensitivity [0.86 (95% CI: 0.74–0.99)] than those published in or before 2020 [0.82 (95% CI: 0.64–1.00)], while specificity (0.64 vs. 0.73) and AUC (0.77 vs. 0.86) showed the inverse pattern. Studies employing a diagnostic cut-off value >15.44 reported lower sensitivity (0.72 vs. 0.92) but higher specificity (0.76 vs. 0.59) and AUC (0.80 vs. 0.75) than those using lower thresholds. Study population stratification revealed that studies focusing on non-procedural AF phenotypes had the highest sensitivity [0.91 (95% CI: 0.74–1.00)] and AUC [0.94 (95% CI: 0.91–0.96)], followed by those conducted in AF recurrence (sensitivity: 0.86; specificity: 0.60; AUC: 0.83) and new-onset AF (sensitivity: 0.80; specificity: 0.73; AUC: 0.83).

**Table 2:** Meta-regression analysis and subgroup analysis.

Parameter	Category	Number of studies	Sensitivity			Pb	Specificity			Pb	Joint model analysis			AUC
			Pooled sensitivity (95% CI)	I^2^%	Pa		Pooled specificity (95% CI)	I^2^%	Pa		LRTchi2	Pc	I^2^ (%) (95% CI)	
Sample size	>600	4	0.71 (0.49–0.93)	92.77	0.00	0.02	0.78 (0.72–0.84)	92.66	0.00	0.72	8.31	0.02	76 (47–100)	0.82 (0.78–0.85)
	≤600	6	0.90 (0.83–0.98)	79.52	0.00		0.60 (0.52–0.68)	89.96	0.00					0.74 (0.70–0.78)
Ethnicity	Asian	4	0.80 (0.60–0.99)	95.94	0.00	0.29	0.64 (0.51–0.77)	97.37	0.00	0.07	2.43	0.30	18 (0–100)	0.77 (0.73–0.80)
	Caucasian	6	0.87 (0.76–0.99)	60.58	0.00		0.71 (0.61–0.80)	89.67	0.00					0.79 (0.75–0.82)
Male proportion	>70%	3	0.79 (0.55–1.00)	69.34	0.03	0.41	0.75 (0.64–0.86)	83.44	0.00	0.57	1.77	0.41	0 (0–100)	0.83 (0.77–0.89)
	≤70%	7	0.87 (0.76–0.98)	95.23	0.00		0.65 (0.56–0.74）	95.45	0.00					0.78 (0.74–0.81)
Age	>60	5	0.85 (0.70–0.99)	97.73	0.00	0.67	0.66 (0.55–0.77)	97.53	0.00	0.08	0.48	0.79	0 (0–100)	0.77 (0.73–0.81)
	≤60	5	0.85 (0.70–0.99)	71.06	0.01		0.71 (0.60–0.81)	90.22	0.00					0.84 (0.80–0.87)
Publication year	>2020	6	0.86 (0.74–0.99)	96.39	0.00	0.98	0.64 (0.54–0.74)	96.60	0.00	0.02	1.37	0.50	0 (0–100)	0.77 (0.73–0.80)
	≤2020	4	0.82 (0.64–1.00)	49.67	0.12		0.73 (0.63–0.84)	86.75	0.00					0.86 (0.83–0.89)
Cut off	>15.44	5	0.72 (0.54–0.90)	90.44	0.00	0.01	0.76 (0.69–0.83)	93.98	0.00	0.83	7.65	0.02	74 (42–100)	0.80 (0.77–0.84)
	≤15.44	5	0.92 (0.85–0.99)	82.90	0.00		0.59 (0.50–0.69)	90.80	0.00					0.75 (0.71–0.79)
Study population	New-onset AF	4	0.80 (0.72–0.87)	61.58	0.05	/	0.73 (0.65–0.81)	86.96	0.00	/	/	/	/	0.83 (0.79–0.86)
	AF recurrence	4	0.86 (0.81–0.92)	78.43	0.00	/	0.60 (0.51–0.70)	96.24	0.00	/	/	/	/	0.83 (0.80–0.86)
	Non-procedural AF	2	0.91 (0.74–1.00)	94.37	0.00	/	0.73 (0.58–0.89)	97.46	0.00	/	/	/	/	0.94 (0.91–0.96)

95% CI, 95% confidence interval; Pa, value for heterogeneity within each subgroup; Pb, value for heterogeneity between subgroups with meta-regression analysis; Pc, value for joint model estimates; AUC, area under the curve.

#### 3.2.6 Sensitivity analysis

**Figure 8:**
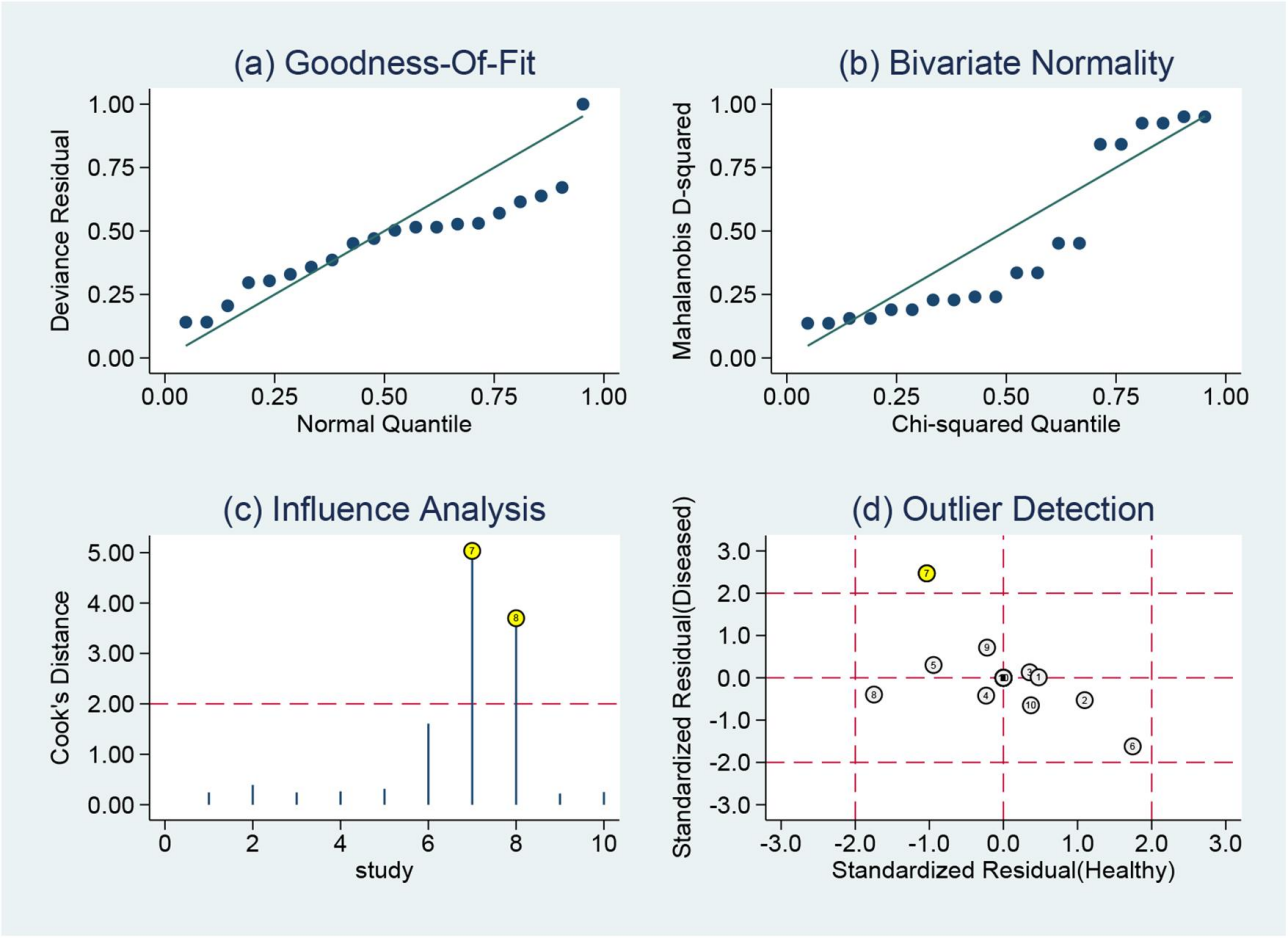
Sensitivity analysis results of MHR in diagnosing AF: **(a)** goodness of fit, **(b)** bivariate normality, **(c)** influence analysis, and **(d)** outlier detection.

Goodness-of-fit and bivariate normality tests confirmed the suitability and reliability of using a bivariate mixed-effects model for meta-analysis (Figures 8a,b). Sensitivity analysis identified two studies (28, 30) that strongly influenced study weights (Figure 8c), while outlier detection revealed one aberrant study (28) (Figure 8d). Subsequent reanalysis was performed after excluding these two studies (Supplementary Table S1). Results showed that pooled sensitivity decreased from 0.85 to 0.80, whereas pooled specificity increased from 0.68 to 0.73. Additionally, pooled +LR rose from 2.67 to 2.94, and the pooled −LR increased from 0.22 to 0.27. The pooled DOR declined from 11.94 to 10.91. Finally, the AUC of the SROC curve improved from 0.80 to 0.81. The effect sizes from the reanalysis remained relatively robust relative to the pooled results before excluding the two studies, indicating that the findings of this study were stable.

#### 3.2.7 Publication bias

**Figure 9:**
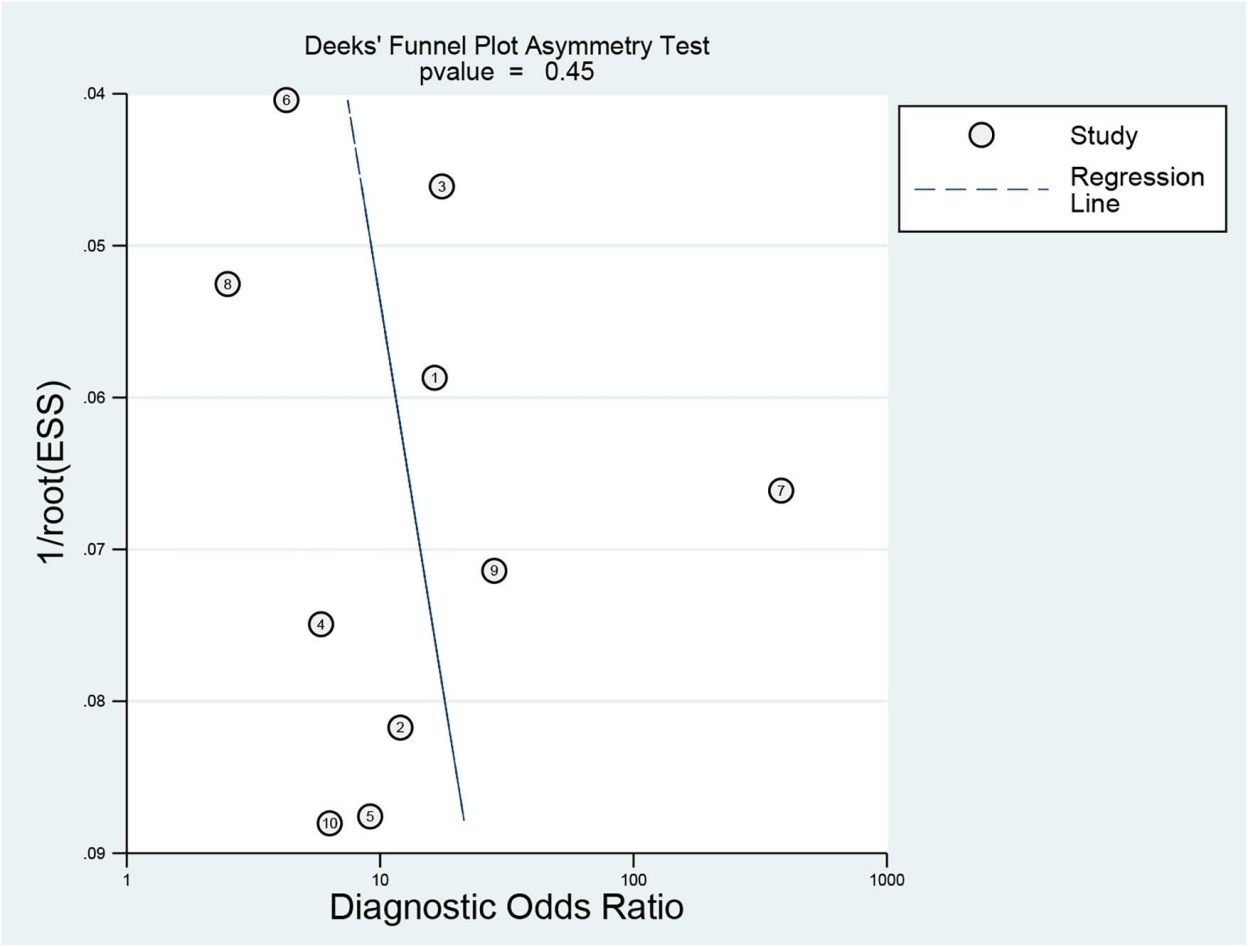
Funnel plot for publication bias assessment of included studies.

Linear regression analysis was used to assess funnel plot asymmetry. The Deeks' funnel plot was symmetrical, indicating no evidence of publication bias (*P* = 0.45) (Figure 9).

## 4 Discussion

To the best of our knowledge, this meta-analysis is the first to evaluate the association between MHR and AF, incorporating data from 5,499 patients across thirteen studies. The findings indicate that elevated MHR is independently associated with a 21% increased risk (OR, 1.21; 95% CI: 1.11–1.31) of developing AF. Regarding diagnostic accuracy, the pooled sensitivity and specificity of the MHR for predicting AF were 0.85 and 0.68, respectively, with an area under the SROC curve of 0.80. According to this result, the MHR exhibits moderate diagnostic accuracy for AF risk prediction. However, given the significant heterogeneity across the different studies, caution is warranted in generalizing and interpreting these findings.

AF is one of the most prevalent arrhythmias and poses a significant challenge in daily clinical practice. Major adverse outcomes associated with AF include stroke, heart failure, sudden cardiac arrest, cognitive impairment, depression, and increased mortality. Although the precise pathogenesis of AF remains incompletely understood, current evidence indicates that inflammation and oxidative stress are central to its onset, persistence, and progression (4). Atrial tissue biopsies from AF patients show inflammatory infiltrates and oxidative damage, which are absent in non-AF control subjects (33). Moreover, AF can induce inflammation during atrial remodeling by recruiting inflammatory cells that release ROS, cytokines, and growth factors, resulting in increased extracellular matrix deposition and subsequent pathological atrial remodeling (34). The interaction between oxidative stress and inflammation further amplifies matrix remodeling and exacerbates atrial fibrosis (35). Activated inflammatory cells and mediators contribute to endothelial dysfunction and platelet activation, fostering a prothrombotic state and linking inflammation to thrombosis in AF (10). Previous studies have indicated that drugs with immunomodulatory properties, including glucocorticoids, HMG-CoA reductase inhibitors (statins), and angiotensin-converting enzyme (ACE) inhibitors, may be effective strategies for intervening in AF progression (36).

WBC count and its subtypes are established markers of inflammation. Monocytes, comprising approximately 2% to 8% of total WBCs, are essential components of the innate immune system. They play a critical role in the development and progression of cardiovascular diseases by contributing to atherosclerotic plaque formation and promoting plaque instability (15). As key effector cells in the inflammatory response, monocytes are pivotal mediators in AF. Although the precise mechanisms remain incompletely defined, several potential pathways have been identified. Monocytes activate the inflammatory cascade by adhering to injured vascular endothelial cells (37) and inducing the production of pro-inflammatory cytokines, such as IL-1, IL-6, TNF-α, transforming growth factor-α, platelet-derived endothelial growth factor, macrophage colony-stimulating factor, and insulin-like growth factor. These cytokines intensify local and systemic inflammation, ultimately resulting in atrial tissue damage (20, 37–39). Monocytes also generate ROS and reactive nitrogen species (RNS), which exacerbate oxidative stress and induce injury and apoptosis in atrial myocytes (40). Additionally, they secrete factors including transforming growth factor-β (TGF-β) and matrix metalloproteinases (MMPs), promoting atrial fibroblast proliferation and collagen deposition, thereby initiating atrial structural remodeling (41). Furthermore, monocytes can alter ion channel expression and function, disrupting the electrophysiological stability of atrial myocytes (42). Fontes et al. demonstrated that patients who developed AF after cardiac surgery exhibited heightened leukocyte activation, with monocyte activation particularly pronounced (43).

Dyslipidemia is a recognized risk factor for both atherosclerotic vascular disease and AF. HDL-C is the only lipoprotein inversely correlated with atherosclerosis and is distinguished by its anti-inflammatory and antioxidant properties. HDL-C inhibits the expression of endothelial adhesion molecules, prevents monocyte infiltration into the arterial wall, reduces the release of inflammatory factors, and decreases oxidative stress (44). Conversely, low HDL-C levels are associated with increased inflammation. Statins and experimental agents can enhance HDL-C's anti-inflammatory effects by inhibiting macrophage inflammatory activity (37). HDL-C also facilitates reverse cholesterol transport, which reduces lipid deposition in atrial tissue, suppresses fibrosis and extracellular matrix remodeling (45), promotes cellular cholesterol efflux, regulates vascular inflammation and vasomotor function, and reduces thrombus formation (46). Additionally, HDL modulates ion channel function and calcium homeostasis, thereby altering atrial electrophysiological properties and increasing susceptibility to AF (47). Reduced HDL-C levels compromise these protective effects, further contributing to AF development and progression (27). Epidemiological studies support the association between HDL-C and AF risk. For instance, an extensive cohort study of 28,449 AF-free individuals (mean follow-up: 4.5 ± 2.7 years) found that low HDL-C was independently associated with new-onset AF in women (48). Similarly, a cross-sectional analysis of 13,724 participants (including 708 with AF) demonstrated an inverse association between higher HDL-C levels and AF risk in individuals younger than 75 years (49).

The MHR reflects the balance between inflammation and oxidative stress, which is driven by the pro-inflammatory activity of monocytes and the anti-inflammatory and antioxidant properties of HDL-C. Many studies have established MHR as a significant predictor of both early and late recurrence of AF following catheter ablation (20, 25, 26, 29–31). Moreover, MHR is associated with AF occurrence, particularly in patients with new-onset AF after CABG or PCI (21–24, 27, 28, 32). In addition, a study by Deng et al. reported that elevated MHR is associated with the presence of left atrial appendage thrombus (LAAT) and spontaneous echo contrast (SEC) in patients with non-valvular AF, suggesting that MHR can serve as an independent predictor of LAAT or SEC in this population (50). Moreover, the degree of inflammation in AF patients is positively correlated with MHR levels (27, 51). Elevated MHR indicates a shift toward a net pro-remodeling state, characterized by increased inflammation, enhanced oxidative stress, and a higher fibrotic tendency (52). These factors collectively disrupt atrial electrophysiological and structural homeostasis, positioning MHR as a sensitive biomarker of the systemic pathological environment that develops during AF progression. Future research should explore specific inflammatory and oxidative stress pathways involved in AF development and progression, as well as the interactions between MHR and these pathways, thereby providing a foundation for developing targeted therapeutic strategies to reduce the incidence of AF.

This study aggregated data from various AF clinical scenarios to assess the predictive value of MHR, based on three primary rationales. First, inflammation and oxidative stress are core pathological mechanisms underlying AF, supporting the theoretical applicability of MHR across different scenarios. Second, all included studies utilized unified AF diagnostic criteria consistent with current clinical guidelines. Third, this approach aimed to systematically validate the broad clinical utility of MHR. However, this pooling introduced significant heterogeneity, which was not attributable to threshold effects (Spearman correlation, *P* = 0.067). Sample size significantly influenced diagnostic sensitivity: studies with larger samples (>600 patients) demonstrated lower pooled sensitivity than smaller studies (0.71 vs. 0.90, *P* = 0.02), suggesting that small-sample studies may overestimate sensitivity due to selection bias, whereas larger studies with rigorous designs provide more reliable results. The MHR cut-off value also affected sensitivity: a cut-off >15.44 was associated with lower sensitivity than a cut-off of 15.44 or less (0.72 vs. 0.92, *P* = 0.01), reflecting a diagnostic trade-off between reducing misdiagnosis and increasing missed diagnoses. The lack of a standardized MHR cut-off across studies further contributed to heterogeneity. Publication year primarily impacted specificity, with studies published after 2020 exhibiting lower specificity (0.64 vs. 0.73, *P* = 0.02), potentially due to more complex study populations (e.g., higher comorbidity prevalence) or the use of more lenient cut-offs, while sensitivity remained stable, confirming MHR's consistent ability to identify AF risk. This study thoroughly evaluated multiple potential sources of heterogeneity and confirmed subgroup analysis results using a joint model. Nevertheless, limited number of studies in some subgroups may have reduced statistical power, necessitating cautious interpretation. Additional unexplained heterogeneity may arise from differences in MHR detection methods, population characteristics, and clinical settings. Specifically, distinct inflammatory profiles and atrial remodeling processes associated with different AF phenotypes and these clinical contexts may also influence MHR's predictive performance, representing a key contributor to heterogeneity.

Several hematological indices, including the neutrophil-to-lymphocyte ratio (NLR) (53), lymphocyte-to-monocyte ratio (LMR) (54), and systemic immune–inflammation index (SII) (55), have demonstrated predictive value in patients with AF. A key clinical question is which of these indices offers the most substantial predictive value for AF. Kutlay et al. conducted a retrospective study to investigate the relationship between hematological indices and AF risk, and found that MHR (AUC = 0.797) was significantly superior to NLR (AUC = 0.646), LMR (AUC = 0.659), and SII (AUC = 0.662) in predicting AF (28). In another retrospective study, Karataş et al. reported that MHR (AUC = 0.843) also exhibited greater predictive power for new-onset AF compared to NLR (AUC = 0.642) (21). These findings indicate that MHR provides a distinct advantage over other hematological markers in predicting AF.

Chen et al. recently conducted a meta-analysis of 21 studies involving 63,687 patients with AF to assess the prognostic value of traditional inflammatory markers derived from neutrophils, lymphocytes, and platelets—specifically the platelet-to-lymphocyte ratio (PLR), NLR, and SII (56). This analysis confirmed that elevated NLR is significantly associated with the risk of adverse outcomes in AF patients, including all-cause mortality, stroke, AF recurrence, and left atrial thrombosis. However, evidence regarding PLR and SII remains limited and inconsistent across studies. By comparison, the present study examines the association between MHR, an emerging inflammatory-metabolic marker, and the risk of AF onset. While Chen et al. clarified the prognostic role of NLR in AF, the current findings emphasize the potential value of MHR in AF risk prediction. These studies are complementary in their research focus. Both analyses acknowledge limitations related to the number of included studies, methodological heterogeneity, and lack of standardization. Notably, the present results highlight the importance of metabolic factors in the comprehensive assessment of AF-related inflammatory markers. Collectively, these findings enhance understanding of the pathophysiological mechanisms underlying AF and provide a foundation for optimizing clinical risk stratification and management of AF patients.

This meta- analysis identifies a significant association between MHR and AF risk. Although MHR demonstrated high diagnostic sensitivity for AF, its moderate specificity and limited positive likelihood ratios indicate it is better suited as a screening or complementary biomarker rather than a standalone diagnostic tool. As AF is partially preventable (57), the development of novel risk-stratification biomarkers is essential for early identification and intervention. In clinical practice, MHR is particularly suitable for initial AF risk stratification in community or primary care settings because of its simplicity and cost-effectiveness. It helps identify individuals at high risk of AF, supporting timely lifestyle interventions or targeted electrocardiographic screening. However, in settings with low AF prevalence, such as the general population or routine outpatient clinics, the relatively high false-positive rate may result in unnecessary investigations, patient anxiety, and increased healthcare costs. Therefore, MHR is more appropriately applied to high- risk subgroups, such as patients after cardiac surgery or those with metabolic abnormalities. Preoperative monitoring of MHR can effectively predict AF occurrence or recurrence in patients undergoing catheter ablation or perioperative cardiac surgery, thereby informing decisions about intensive cardiac monitoring or prophylactic antiarrhythmic therapy. Combining MHR with established clinical risk parameters, such as the APPLE score, has been shown to further improve predictive accuracy for postoperative AF recurrence after catheter ablation (29). Whether the combination of MHR and the CHA_2_DS_2_-VASc score provides incremental value for stroke risk prediction in patients with non-valvular AF remains unresolved. The incremental prognostic value of MHR over established AF risk stratification scores is currently hypothetical. It requires prospective validation using quantitative metrics such as net reclassification improvement, integrated discrimination improvement, or decision curve analysis.

Several potential limitations should be considered in this meta-analysis. First, most included studies were retrospective, introducing unavoidable selection bias and residual confounding. Second, MHR cut-off values varied substantially across studies, likely reflecting differences in laboratory units and measurement methodologies. This variability may have impeded inconsistent diagnostic outcomes across institutions and hindered the standardized clinical implementation of MHR. Third, the QUADAS-2 assessment indicated that non-predefined or data-driven cut-off values may lead to overfitting, potentially overestimating diagnostic performance and compromising the robustness and generalizability of the pooled results. Fourth, the analysis relied solely on a single MHR measurement. Integrating MHR with additional biomarkers (e.g., hs-CRP, IL-6) and clinical scores (APPLE, CHA_2_DS_2_-VASc) using machine- learning models, as well as dynamic continuous MHR monitoring, may provide a more comprehensive assessment of disease status and improve predictive accuracy. Fifth, all included studies were conducted in China and Turkey, which may introduce regional selection bias. Future research should focus on multicentre prospective studies, standardisation of MHR detection protocols, development of universal diagnostic thresholds, and incorporation of dynamic monitoring to enhance clinical utility.

## 5 Conclusion

In conclusion, this meta-analysis demonstrates that elevated MHR is independently associated with a 21% increase in the risk of AF (OR = 1.21; 95% CI, 1.11–1.31; *P* < 0.001). The pooled sensitivity and specificity of MHR for AF prediction are 0.85 and 0.68, respectively, with an area under the SROC curve of 0.80, which suggests moderate diagnostic accuracy for AF risk prediction. MHR is more appropriately positioned as a screening or complementary biomarker rather than a standalone diagnostic tool for AF. Notably, the diagnostic performance of MHR may differ by AF phenotype and clinical context. However, given the limited number of included studies, lack of standardized measurement methods, and absence of unified reference ranges, future research should prioritize large-scale prospective studies, standardized methodologies, and the establishment of uniform cut-off thresholds. Beyond validating the predictive accuracy of MHR for AF risk, further efforts should aim to promote its routine implementation in clinical practice.

## Data Availability

The original contributions presented in the study are included in the article/Supplementary Material, further inquiries can be directed to the corresponding authors.

## Supplementary material

Supplementary Figure S1 Forest plot depicting the likelihood ratio of the MHR in predicting AF.

Supplementary Figure S2 Forest plot depicting the diagnostic score and diagnostic odds ratio of the MHR in predicting AF.
