# Supplementary material for: Predictive value of the monocyte-to-high-density lipoprotein cholesterol ratio in atrial fibrillation: a meta-analysis
Source: Front Cardiovasc Med. 2026 Feb 2;13:1620841. doi: 10.3389/fcvm.2026.1620841 (PMC12907383; doi:10.3389/fcvm.2026.1620841)
Supplement: Supplementary Figure S1 — Forest plot depicting the likelihood ratio of the MHR in predicting AF. [file Datasheet1.docx]

Supplementary Materials

**Table S1.** Diagnostic performance after removing the two-weight types of research.

| Diagnostic performance | Pooled value | Removed Kutlay (28) and Aimaitijiang (30) |
| --- | --- | --- |
| Sensitivity | 0.85 (95% CI: 0.71−0.93) | 0.80 (95% CI: 0.69−0.88) |
| Specificity | 0.68 (95% CI: 0.60−0.75) | 0.73 (95% CI: 0.67−0.78) |
| +LR | 2.67 (95% CI: 2.16−3.30) | 2.94 (95% CI: 2.55−3.40) |
| −LR | 0.22 (95% CI: 0.12−0.42) | 0.27 (95% CI: 0.18−0.42) |
| DOR | 11.94 (95% CI: 5.90−24.17) | 10.91 (95% CI: 7.10−16.76) |
| AUC | 0.80 (95% CI: 0.76−0.83) | 0.81 (95% CI: 0.77−0.84) |
| +LR, positive likelihood ratio; −LR, negative likelihood ratio; DOR, diagnostic odds ratio; AUC, area under the curve. | | |


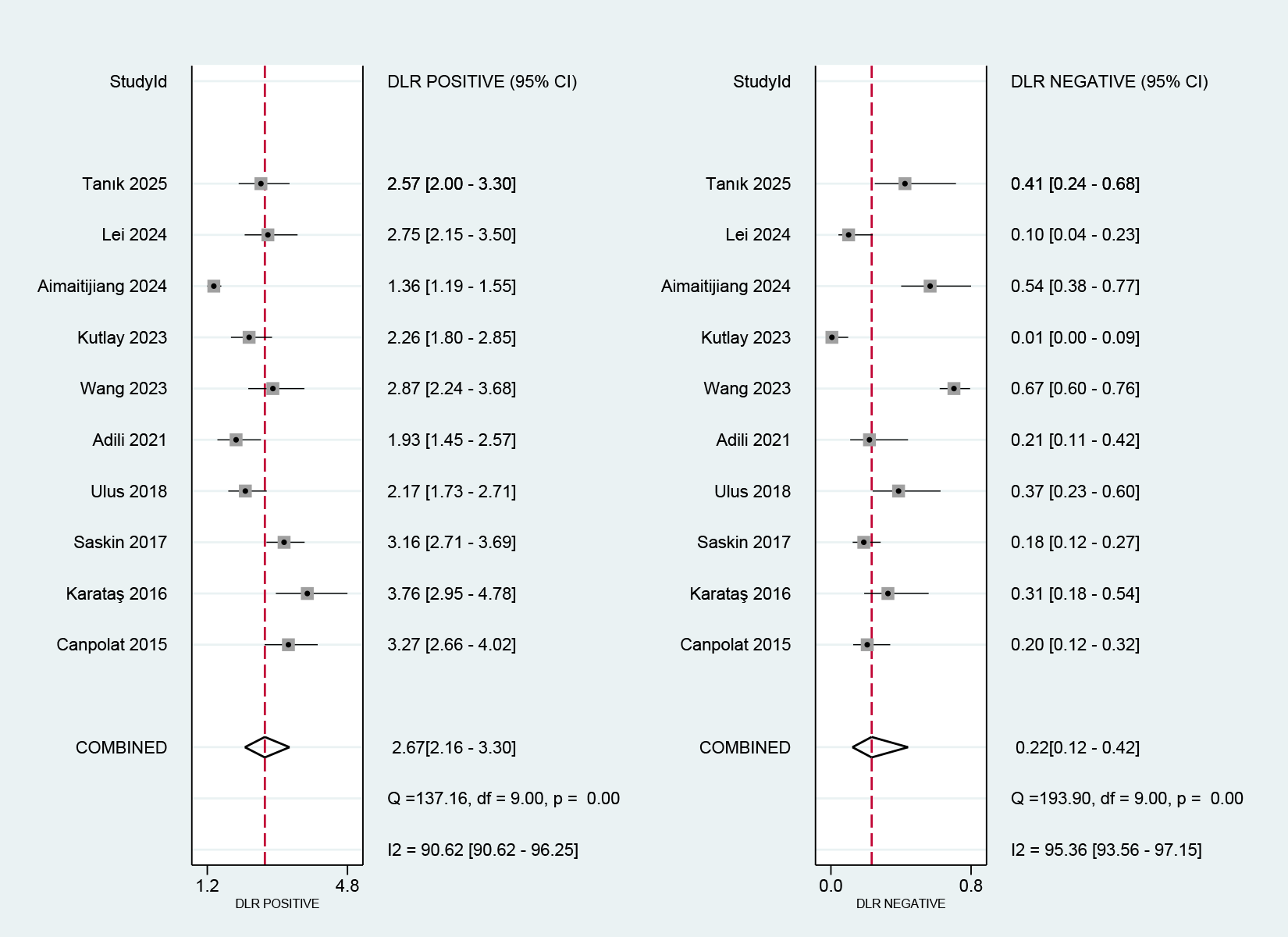


**Figure S1:** Forest plot depicting the likelihood ratio of the MHR in predicting AF.


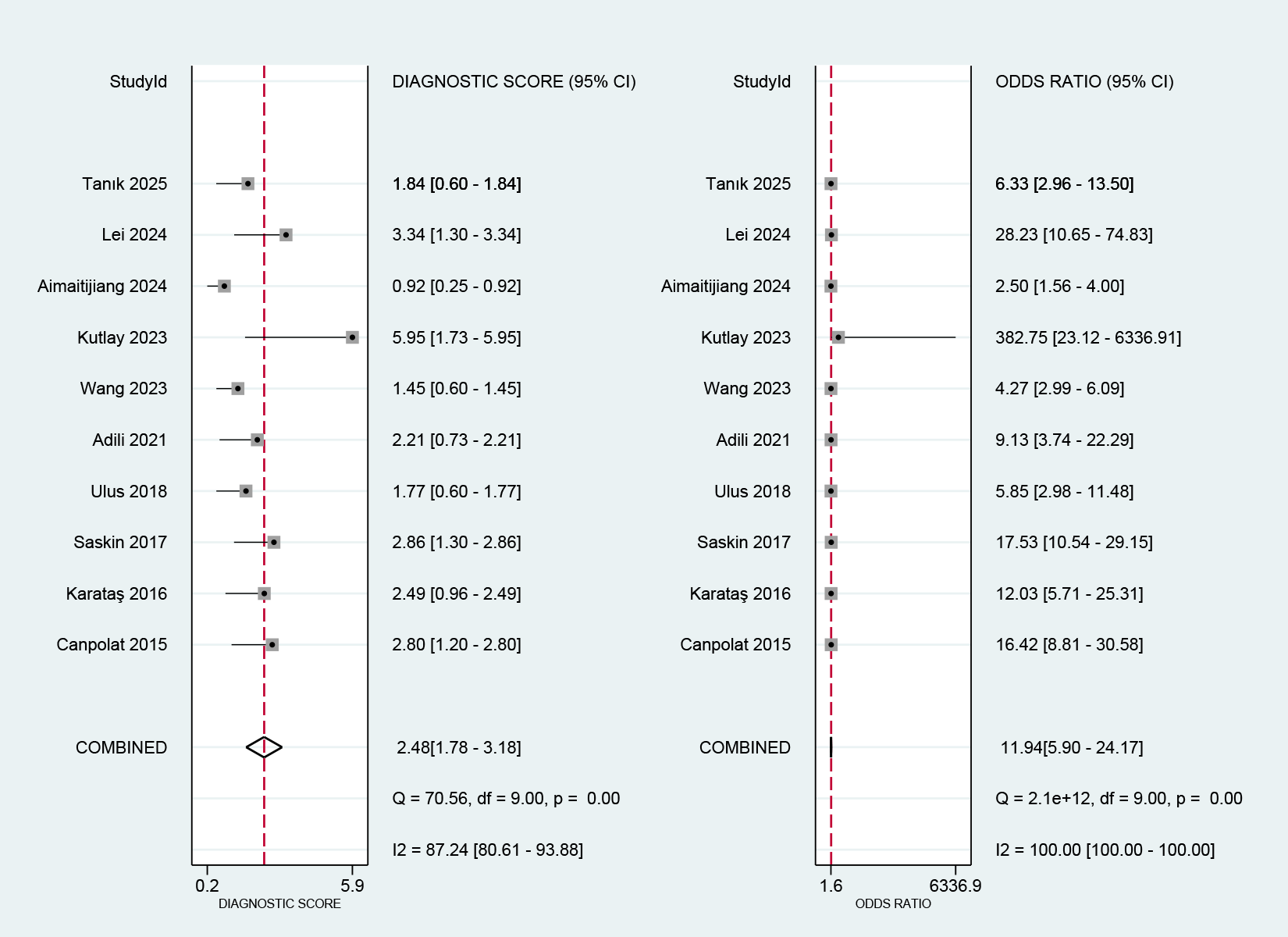


**Figure S2:** Forest plot depicting the diagnostic score and diagnostic odds ratio of the MHR in predicting AF.
